# Molecular Mechanisms of Iron Mediated Programmed Cell Death and Its Roles in Eye Diseases

**DOI:** 10.3389/fnut.2022.844757

**Published:** 2022-04-05

**Authors:** Jie Zhang, Shuai Sheng, Wenting Wang, Jiazhen Dai, Yifan Zhong, Jiantao Ren, Keke Jiang, Shuchan Li, Xiaoyan Bian, Lei Liu

**Affiliations:** ^1^Department of Public Health, Weifang Medical University, Weifang, China; ^2^Department of Ophthalmology, Weifang Eye Hospital, Weifang, China; ^3^Department of Ophthalmology, The First Affiliated Hospital of China Medical University, Shenyang, China; ^4^Department of Ocular Surface, Baotou Chaoju Eye Hospital, Boatou, China; ^5^Department of Ophthalmology, Guangdong Provincial People’s Hospital, Guangdong Eye Institute, Guangdong Academy of Medical Sciences, Guangzhou, China

**Keywords:** eye diseases, cell death, iron, ferroptosis, lipid peroxidation

## Abstract

Ferroptosis, a newly identified, iron-dependent type of programmed cell death, is active in several diseases, such as heart disease, brain damage, and cancer. Its main characteristics commonly involve excess iron accumulation, elevated lipid peroxides and reactive oxygen species, and reduced levels of glutathione and glutathione peroxidase 4 levels. The effects of ferroptosis in eye diseases cannot be underestimated, with ferroptosis becoming a research target in ocular disorders and emerging evidence from a series of *in vivo* and *in vitro* researches into ferroptosis revealing its role in eye conditions. However, no report provides comprehensive information on the pathophysiology of ferroptosis in eye diseases and its possible treatments. In the current review, we present an up-to-date overview of ferroptosis biology and its involvement in the pathological processes of ocular diseases. Furthermore, we pose several outstanding questions and areas for future research in this topic. We deem ferroptosis-associated cell death a pivotal new field of scientific study in ocular diseases and consider it a new therapeutic target in the treatment of some eye disorders.

## Introduction

There are many kinds of cell death, which differ according to morphology, including apoptosis, autophagy, and necrosis. Apoptosis and autophagocytic cell death are modulated by signaling pathways, whereas necrosis is classified as “accidental cell death” and is considered passive and not regulated by signaling pathways (1–3). Ferroptosis is a type of iron-dependent programmed cell death that is regulated by specific genes and is distinct from apoptosis, necroptosis, and autophagy. In addition, ferroptosis is different from common cell death in terms of morphology. It can induce mitochondrial membrane crumpling and increase mitochondrial membrane density (4). Abnormal iron metabolism, lipid peroxidation, and accumulation of polyunsaturated fatty acid phospholipids can all trigger ferroptosis. Additionally, iron metabolism, lipid peroxidation, and polyunsaturated fatty acid phospholipids metabolism perform imperative moderating role in numerous nutritional imbalance, thus ferroptosis has a link with nutrition metabolism.

Researchers have long observed ferroptosis but classified it as one of the other types of cell death, and even dismissed it as meaningless (5). Early research revealed a relationship between necrocytosis and cellular metabolism. In the early 1970s, glutathione (GSH) depletion was reported during mouse hepatocyte necrosis, and the addition of cysteine or GSH was observed to inhibit such cell death (6). In 2012, Stockwell’s (7) team identified a specific chemical, erastin, in a HT-1080 fibrosarcoma cell model that is capable of regulating voltage-dependent anion channels (VDACs) in mitochondria. Erastin not only plays an antitumor role by regulating VDACs in mitochondria, but also induces ferroptosis in cells. In addition, erastin can inhibit the function of solute carrier family 7 member (SLC7A11), an important component of the Na^+^-independent cystine/glutamate antiporter system [system Xc(–)] and reduce the levels of the raw materials for GSH synthesis in cells. This leads to augmented production of iron-dependent lipid reactive oxygen species (ROS) and induces ferroptosis in tumor cells (7).

In the subsequent years, several studies confirmed that reduced intracellular cysteine content and high GSH consumption played a key role in cell death. It was proven that lipophilic antioxidants can inhibit this mode of cell death, which are the main characteristics of ferroptosis (8). Because intracellular ROS is mainly produced by lipid metabolism, abnormal lipid metabolism leads to ferroptosis (9). However, it was not until 2012 that the Nomenclature Committee on Cell Death recommended that researchers classify ferroptosis based on the molecular basis of the cell death that it gained its own name and attention (5). In the present review, we provide an up-to-date overview of the mechanisms of ferroptosis and discuss current research and future research prospects in the field of ocular diseases.

## Mechanisms of Ferroptosis

**FIGURE 1:**
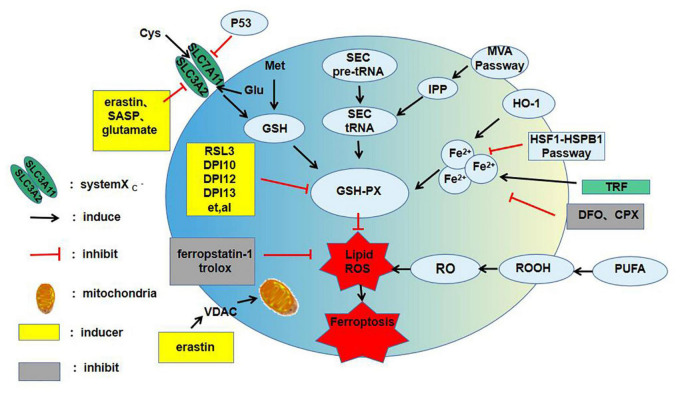
The main mechanism of ferroptosis. ROS, reactive oxygen species; SASP, salicylazosulfapyridine; Cys, cystine; Glu, Glutamate; Met, methionine; GSH, glutathione; GSH-PX, glutathion peroxidase; SEC, selenocysteine; IPP, isopentenyl pyrophosphate; MVA, mevalonic acid; HO-1, haem oxygenase-1; TRF, transferrin; DFO, deferoxamine; CPX, ciclopirox olamine; PUPA, polyunsaturated fatty acid; VDAC, voltage-dependent anion channels; RSL3, ras-selective-lethal compound3; ROOH, lipid hydroperoxide; RO, lipid peroxide; HSF1, heat shock transcription factor 1; HSPB1, heat shock protein 1; DPI, diphenyleneiodonium chloride.

The main characteristics of ferroptosis are: (1) Depletion of GSH and lipid peroxidation; (2) cell death acceleration by iron-dependent intracellular ROS accumulation and iron overload; and (3) cell death inhibition by scavengers of lipid ROS [e.g., ferrostatin 1 (FER-1)] and iron chelators (e.g., ferriamine) (10). Ferroptosis is regulated by different signaling pathways. Most upstream pathways induce ferroptosis by influencing the activity of glutathione peroxidase 4 (GPx-4) (10), and GPx-4 is also the target of many ferroptosis inducers (Figure 1).

### Cystine/Glutamate Antiporter Pathway

System Xc(-) performs an important antioxidant function in the cell. It can extract intracellular glutamate and absorb extracellular cystine to reduce it to cysteine, thereby participating in GSH synthesis (11). GSH can reduce ROS and active nitrogen under the action of GPx-4 to decrease cytotoxicity. Therefore, inhibition of system Xc(-) will result in oxidative damage and even cell death. More and more researchers have revealed that inhibition of system Xc(-) is key to ferroptosis. For example, Dixon et al. (7) established an organotypic hippocampal slice culture and deduced that glutamate-induced cell death might be a signaling pathway of ferroptosis. Further research concluded that the system Xc(-)-mediated cellular uptake of cystine plays a key role in ferroptosis. Inhibition of system Xc(-) leads to compensatory upregulation of SLC7A1L transcription (12), and SLC7A11 transcriptional upregulation was found in salazopyridine- and erastin-induced ferroptosis models. The addition of erastin or high extracellular glutamate levels reduced cysteine, which inhibited system Xc(-) and led to GPx-4 inactivation and ferroptosis induction (13). Thus, the classic oxidative stress pathway can lead to ferroptosis, opening a new avenue for future research.

### Iron Metabolic Pathway

Iron is essential for homeostasis in the human body, with excess iron or its dysregulation associated with the pathological progression of various diseases (14). Iron accumulation can distinguish ferroptosis from other oxidative stress pathways and represents a unique mechanism that induces cell death. Fe^2+^ reduces oxygen to form superoxide radicals, which cause lipid peroxidation through the Fenton reaction and induce ferroptosis in cells (15). Therefore, various factors that cause iron metabolism disorders can subtly affect ferroptosis. The balance of iron storage and use in the human body is mainly affected by ferritin light polypeptide (FTL) and ferritin heavy polypeptide (FTH), and their related genes (16). The expressions of FTL and FTH1 are significantly increased by inhibition of iron response element-binding protein 2 (IREB2), which inhibits ferroptosis induced by erastin (12). The iron involved in lipid peroxidation is derived from unstable intracellular iron pools (17). A specific type of autophagy mediated through the nuclear receptor co-activator 4 (NCOA4, a cargo receptor) called ferritin autophagy degrades ferritin, which in turn increases the size of the unstable iron pool in the cytoplasm to induce ferroptosis (18). Lysosomal degradation occurs through NCOA4 recognition and absorption of ferritin into the autophagosome, resulting in iron release. Therefore, inhibition or induction of iron decreases can be achieved by overexpression or knockout of NCOA4. Acyl-CoA synthetase long-chain family member 4 (ACSL4)-dependent cellular phospholipid processes and the tricarboxylic acid cycle also exhibit increased sensitivity to ferroptosis during iron synthesis and processing.

### Lipid Metabolic Pathway

Ferroptosis is emerging as a new form of iron-dependent regulated cell death (RCD), which is driven by excessive lipid peroxidation inducers in recent years. Furthermore, because lipid peroxidation in cells is regulated by the content and position of polyunsaturated fatty acids (PUFAs), it indirectly determines the degree of ferroptosis (19). The membrane phospholipid family, which contains PUFAs, is the most common lipid family that causes ferroptosis. Toxic lipid ROS are produced by PUFA-containing phospholipids (PUFA-PLs), which are found in the membrane of the whole cell. The common sites of lipid ROS accumulation are the endoplasmic reticulum, mitochondria, and lysosomes (14, 20, 21). Studies of lipid metabolism suggest that phosphatidylethanolamine, which contains arachidonic acid and epinephrine, is a linchpin phospholipid for oxidation and promoting ferroptosis (2). The two iron-dependent lipid peroxidation reactions can be classified as: (1) Non-enzymatic radical chain reactions involving the Fenton process, which produce highly toxic hydroxyl and peroxide radicals; and (2) enzyme-dependent processes involving iron-containing enzymes, such as lipoxygenase (20). Ng et al. (15) found that 15-lipoxygenase-mediated lipid peroxides are downstream of ferroptosis caused by GPx-4 inactivation. The mechanism of the ferroptosis caused by lipid peroxide and the exact location of this phenomenon in cells are currently being actively investigated.

### p53

p53 is a well-known tumor suppressor gene. Studies have shown that inhibition of p53 in tumor cell growth is related to increased ferroptosis sensitivity (22). Mechanistically, p53 induces ferroptosis by directly inhibiting SLC7A11 *via* system Xc(-) or by upregulating recombinant spermidine/spermine N1-acetyltransferase 1 (SAT1) downstream of p53, which is involved in polyamine metabolism (23). In addition to regulating the GPx-4 centered pathway, Chu et al. (24) also found that p53 can activate a new ferroptosis regulatory pathway. They experimentally observed that the function of GPx-4 was not significantly affected during the process of ferroptosis activated by p53. Further experiments showed that inactivation of the arachidonate 12-lipoxygenase (ALOX12) gene on human chromosome 17p13.1, located close to the p53 site, and knockout of an ALOX12 allele is sufficient to inhibit p53-mediated ferroptosis. An ALOX12-mediated ferroptosis pathway was further identified, and this pathway is crucial to the p53-dependent inhibition of cell growth (24).

### Transcription Factor Pathways

**FIGURE 2:**
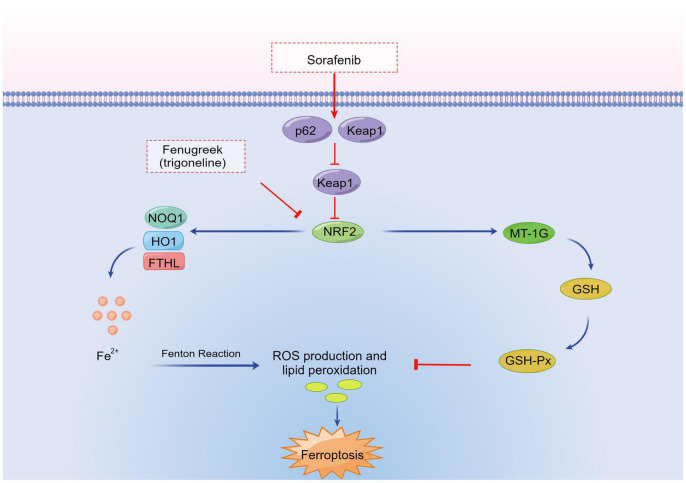
Transcription factor pathways in ferroptosis. NRF2, nuclear factor-erythroid 2-related factor 2; FTHL, frontotemporal hairline; Keap1, Kelch-like ECH-associated protein 1; ROS, reactive oxygen species; MT-1G, metallothionein-1G; HO1, heme oxygenase-1; NOQ1, NAD(P)H/quinone oxidoreductase 1; GSH-Px, glutathione peroxidase; GSH, glutathione.

A major transcription factors, such as nuclear factor-erythroid 2-related factor 2 (NFE2L2, also known as NRF2) (25), are key regulators of antioxidant responses and important in protecting cells from ferroptosis. Sun et al. (25) experimentally identified a ferroptosis signaling pathway with NFE2L2 at its core. Under oxidative stress conditions, NFE2L2 degradation is decreased and a multistep activation pathway is initiated, which inhibits the apoptosis of various cells and promotes chemotherapy resistance. They also showed that, with the addition of a ferroptosis inducer, p62 expression was increased to prevent NFE2L2 degradation and enhance the subsequent accumulation of NFE2L2 nuclei by inactivating Kelch-like ECH-associated protein 1 (Keap1). Further experiments revealed that the p62-Keap1-NFE2L2 pathway inhibits ferroptosis. This pathway participates in iron and ROS metabolism by upregulating several genes, including heme type I fluorosynthase and frontotemporal hairline (FTHL). It enhances the anticell activity of erastin and sorafenib against HCC cells by inhibiting NFE2L2 expression both *in vitro* and *in vivo* (25). Therefore, inhibition of the p62-Keap1-NFE2L2 signal-transduction pathways can significantly enhance the anticancer activity of cells (Figure 2).

### Ferroptosis Regulation

Although the mechanism of ferroptosis is still unclear, some factors that induce or inhibit ferroptosis have recently been found. The inducers are: (1) ACSL4, as a major regulator in the pathophysiology of ferroptosis, which stimulates it by increasing the content of long PUFAs and omega-6 PUFAs in the cell membrane (26); (2) cysteinyl-tRNA synthase (CARS), which stimulates ferroptosis through a transsulfuration pathway (27); (3) type I heme oxygenase, a heme that promotes iron accumulation by degrading enzymes that release iron (28); (4) lipoxygenase (LOX), which stimulates ferroptosis by catalyzing the dioxygen reaction of PUFAs (29); (5) nitrogen oxide metabolites (NOx), which stimulate ferroptosis by increasing ROS production (30); (6) arginine/arginine N1 acetyltransferase, which stimulates ferroptosis by increasing the peroxidation of arachidonic acid (31); and (7) membrane protein transferrin receptor 1 (TfR1), which stimulates ferroptosis by altering iron uptake (32). The inhibitors include: (1) Ferritin, which inhibits ferroptosis by reducing free iron ions (30); (2) heat shock protein 5 (HSPA5), which inhibits ferroptosis by blocking GPx-4 degradation (33); (3) heat shock protein 1 (HSPBL), which protects cells from lipid ROS (30); and (4) mitochondrial ferritin, which increases iron storage and inhibits ferroptosis (34).

## Research Progress Into Ferroptosis in Ophthalmology

The clinical application of ferroptosis is currently mainly being studied in terms of nervous system diseases, tumors, and ischemia/reperfusion injury. However, a series of researches have shown that ferroptosis is also in the development and progression of eye diseases. The following is a brief description of the latest findings in corneal epithelial disease, corneal endothelial cell dysfunction, retinal pigment epithelial (RPE)-associated eye diseases, glaucoma, diabetic retinopathy (DR), retinal ischemia-reperfusion injury (RIRI), retinoblastoma, retinitis pigmentosa (RP), and age-related cataracts (Table 1).

**TABLE 1:** A brief description of the biological role of ferroptosis in its related eye diseases.

Disease	Model (*in vitro* or *in vivo*)	Target biomarker	Biological function of eye diseases	References
DR	*In vivo* (STZ mice)	ROS	Mediates the regulation of angiogenesis	(57)
Corneal epithelial cell	*In vitro* (Human corneal epithelial cell line) *In vivo* (GPx-4^+/+^ and GPx-4^+/^ mice)	GPx-4	Oxidative homeostasis, cell survival, and wound healing	(35)
RPE cell	*In vitro* (ARPE-19 cells)	ROS	SIPS and cell death in human RPE cells	(44)
	*In vitr*o (ARPE-19 cells)	GPx-4	Rescued tert-butyl hydroperoxide (tBH)-induced RPE cell death	(2)
	*In vitro* (SI-induced death of RPE cells)	ROS	Increased oxidative stress	(94)
	*In vivo* monolayer mouse RPE (mRPE) cells from NaIO3-treated retinae	Lipid ROS (LOS)	Oxidative stress-mediated	(47)
Glaucoma	*In vivo* (Male Sprague-Dawley rats)	N-methyl-D-aspartate (NMDA)	Fe^2+^ accumulation in the retinal ganglion cells	(76)
	*In vivo* (Wistar rats)	Adiponectin receptor (AdipoR)	Inhibiting ROS production *via* up-regulation of PGC-1a, Esrra, and TFAM	(95)
RP	*In vivo* (RD10 mice)	VK28 and VAR10303	Partial histologic and functional rescue of cones	(82)
	*In vivo* (wild type and rd10 mice)	GPX	Oxidative damage to the retina	(96)
Cataract	LEGSKO mice	GSH	Genes up-or down-regulated in aged lens epithelial cell	(23)

*DR, Diabetic retinopathy; STZ, streptozotocin; GPx-4, glutathione peroxidase 4; ARPE, human retinal pigment epithelium; PGC-1a, peroxisome proliferator-activated receptor g coactivator-1a; TFAM, mitochondrial transcription factor A; VK28, 5-(4-(2-hydroxyethyl), piperazin-1-yl (methyl)-8-hydroxy-quinoline); VAR10303, 5-(N-methyl-N-propargyaminomethyl)-quinoline- 8-oldihydrochloride; GPx, glutathione peroxidase; SIPS, Stress-induced premature senescence; RP, Retinitis pigmentosa.*

### Cornea Disorders

#### Corneal Epithelial Disease

The cornea is constantly damaged by the external natural environment, and the oxidative stress caused by such damage is closely linked to corneal diseases (32). GPx-4 is one of key anti-oxidant enzymes that converts potentially toxic oxidative damage (lipid peroxidation) products into non-toxic lipid alcohols to maintain the REDOX homeostasis in corneal epithelial cells and promote wound healing and is a central regulator of ferroptosis lipid peroxidation-mediated pathways (23). Decreased expression of GPx-4 leads to cell oxidative stress and cytotoxicity, which culminate in a decreased wound-healing ability of corneal epithelial cells. Sakai et al. (35) revealed an influence of GPx-4 on human corneal epithelial cytotoxicity, lethality, cell activity, and wound healing. Their study involved five experimental groups: Specific knockout of siRNA-transfected human corneal epithelial cells with hydrogenase, GPXL, GPx-4, superoxide dismutase 1 (SOD1), and SOD2; in the control group, human corneal epithelial cells were transfected with siRNA. Lactate dehydrogenase (LDH) activity was used as a cytotoxicity indicator. The results showed that LDH activity was significantly increased only in the GPx-4 and SOD1 knockout groups and that LDH activity was markedly higher in the GPx-4 group than in the SOD1 group. The GPx-4 and control groups were further treated with 100 mL hydrogen peroxide. The LDH activity in the control group was not affected by the hydrogen peroxide, whereas the LDH activity in the experimental group was significantly increased, indicating that GPx-4 plays an essential role in oxidative stress in human corneal epithelial cells.

Furthermore, researchers found that α-tocopherol can prevent apoptosis-inducing factor (Aif) transposition induced by GPx-4 gene knockout. Ferritin-1 ameliorated the decreased cell viability and elevated LDH caused by GPx-4 gene knockout. These results suggest that ferroptosis influences the cytotoxicity and cell death of GPx-4-deficient corneal epithelial cells. They also identified a significant delay in wound healing in GPx-4 knockout corneal epithelial cells and that the addition of α-tocopherol significantly improved the delay in wound healing caused by GPx-4 knockout (35). Here, ferroptosis plays a role in corneal epithelial cell death on account of the oxidative stress caused by GPx-4 deficiency. These studies suggest that ferroptosis inhibition can help to protect corneal epithelial cells.

#### Corneal Endothelial Cell Dysfunction

Corneal endothelial cells (CEnCs) are a layer of a single hexagonal-shaped cells adherent to the Descemet’s membrane in the innermost cornea layer (36, 37). Fuchs’ endothelial corneal dystrophy (FECD) is a typical feature of posterior corneal dystrophy. It is a nutritional disorder characterized by progressive damage to the corneal endothelium and the eventual development of corneal endothelial decompensation, which usually causes low visual acuity. CEnCs promote the development of FECD by increasing apoptosis, thereby elevating sensitivity to oxidative stress. The transcription factor NFE2L2, a regulator of ferroptosis, is downregulated in FECD (38). Lovatt1 et al. (39) demonstrated that expression of the redox sensor, peroxiredoxin 1 (PRDX1) is selectively atrophy from CEnCs in patient with FECD. This was linked to a loss of NFE2L2 expression. The authors established that the ferroptosis inhibitor FER-1 decreases lipid peroxidation and RCD in CEnCs. Ferroptosis inhibitors are being explored as an alternative strategy to a new treatment for FECD. However, no association has yet been found with lipid peroxidation, loss of NFE2L2, and ferroptosis in human CEnCs.

### Retinal Pigment Epithelial-Associated Eye Diseases

Age-related macular degeneration (AMD), as an irreversible neurodegenerative disorder of the macular tissue, is accompanied by progressive vision loss, which gravely affects quality of life and is the main cause of blindness in elderly people in developed countries (40). Oxidative stress and free radical damage are thought to be the main causes of RPE cell damage and AMD progression, and advanced AMD is also related to iron overload in RPE/Bruch membrane (41). 4-hydroxy-2-nonenal (4-HNE) is a signaling molecule that stimulates gene expression and cell survival at physiological levels. 4-HNE has cytotoxic effects that inhibit gene expression and promote cell death at abnormally high levels, *via* the end-products of lipid peroxidation (42). Sharma et al. reported that 4-HNE participates in p53-mediated signaling by phosphorylating p53, inducing activation and nuclear aggregation and leading to apoptosis (43). However, it has not been reported whether 4-HNE can induce apoptosis of RPE cells.

GSH is the most potent antioxidant in RPE cells, with high levels in the retina and RPE cells (44). Moreover, reduced GSH leads to cell death. Sun et al. (44) used the standard *in vitro* model of AMD to prove that GSH reduced the RPE death induced by ferroptosis and autophagy and observed that ferroptosis inhibitors inhibited primary RPE cell death more effectively than apoptosis or necrosis inhibitors. Another study further demonstrated that autophagy-related ferroptosis induced by GSH reduction (45). Totsuka et al. (46) additionally showed that ferroptosis plays a fundamental role in degeneration/death of RPE. They compared the effects of cysteine proteolytic enzyme inhibitors, necrosis inhibitors, and ferroptosis inhibitors on the activity of human retinal pigment epithelial cell line (ARPE-19) after exposure to 500 μmol/L tert-butyl hydroperoxide (TBH) *in vitro*. The results showed that all three inhibitors improved cell viability at low concentrations of TBH. However, with high concentrations of TBH, the ferroptosis inhibitors improved the viability of ARPE-19 cells. The same results were obtained with primary cultured human fetal RPE cells. We further assessed total ROS levels and lipid peroxidation levels in ARPE-19 cells exposed to 500 μmol/L TBH. The total ROS level detected was upregulated at 3 and 6 h, but the upregulation was significantly inhibited by ferroptosis inhibitor. Similarly, the degree of lipid peroxidation evaluated by BODIPY staining was consistent with the change in the total ROS level. A significant reduction in GSH levels was seen. Moreover, it was observed that the intracellular Fe^2+^ level increased in TBH-treated ARPE-19 cells but that there was no significant change in Fe^2+^ after pretreatment with FER-1 or deferoxamine (DFO) (46). Tang et al. (47) demonstrated that, in the sodium iodate-induced oxidative stress model, regulation of ferroptosis by heme oxygenase-1 (HO-1) was the main pathological process underlying RPE cell degeneration. Ferroptosis of RPE cells can be significantly blocked by knockout of HO-1 or inhibition of HO-1 overexpression with the HO-1 inhibitor ZnPP, with good therapeutic effects. Hence, inhibition of ferroptosis might be a new target for dry AMD (46).

In neovascular AMD, RPE cells generate vascular endothelial growth factor (VEGF) when they are under hypoxia or oxidative stress (48–50). A rodent model of choroidal neovascularization (CNV) was established to determine the protein expression level of SLC7A11, a regulator of ferroptosis, and VEGF during disease progression and assessed the role of SLC7A11 in laser-induced CNV model. They examined protein expression of enzymes related to oxidative stress and Fe^2+^. Their findings indicated that ferroptosis in CNV induced by laser was accompanied by increased the content of Fe^2+^ and expression of GPx-4 in the isolated RPE/choroid complexes. Meanwhile, they confirmed the SLC7A11 expression in the ARPE-19 cell line and the roles of its inhibitors on cell viability and function as well as the extent of lipid peroxidation, with the results suggesting that SLC7A11 can possibly represent a compelling target for therapeutic intervention in patients with neovascular AMD.

Danon’s disease (DD) is an X-linked single-gene disease in which vision loss can begin at a young age. Patients with DD have a characteristic disordered RPE arrangement in the retina (23). In addition, some studies have found that a primary deficiency of the Lysosome-associated membrane protein-2 (LAMP-2) may be the cause of the visual impairment in DD patients. This mutation not only induces lipodystrophy of cone and rod cells (23), but also damages the tight junctions of RPE cells (51). Ferroptosis may be one of the important cell death mechanisms of LAMP-2 deficiency and ROS exposure. Apoptosis inhibitors or necrostatin 1 are unable to prevent LAMP-2 knockdown cell death induced by ROS in human RPE-19 cells, but the iron-chelating agent DFO can prevent LAMP-2 knockdown cell death (52). Similarly, 2,2′-bipyridine, a fast-acting iron chelator that acts similarly to DFO, protects LAMP-2 knockdown cells from tributyl phosphate (THP)-induced cell death in human RPE-19 cells and in primary fetal retinal pigment epithelium (52). The effects of such iron-chelating agents are consistent with the results of ROS-induced ferroptosis studies (46). This increased sensitivity to ferroptosis inducers is further supported by the high sensitivity of LAMP-2 mutated cells to ferroptosis (53). Cysteine levels are decreased in the cytoplasm of ARPE-19 cells with LAMP-2 gene deletion. Because cysteine is a precursor of GSH, GSH levels are lower in cells with LAMP-2 gene deletion. In addition, cysteine and glutamine pretreatment for 24 h significantly reduces THP-induced LAMP-2 knockdown cell death (53). These results further confirm that, in the retina of DD patients, deletion of the LAMP-2 gene can cause ROS-induced ferroptosis of RPE cells through cysteine reduction and that the retina of DD patients can be protected by iron-chelating agents and cysteine supplementation.

### Diabetic Retinopathy

Diabetic retinopathy (DR), which can culminate in irreversible blindness, is the most serious diabetic eye disease (54). Neuronal apoptosis and reactive glial degeneration have recently been identified as early alterations in DR, but the exact cause of the neurodegeneration has not been found (55). Because the nerves and blood vessels of human brain and eyes share the same origin, studies have found that the abnormal high phosphorylation of tau-related proteins, which are characteristic pathological changes of Alzheimer’s disease (AD), leading to a gradual loss of neurons, is tightly connected with the occurrence and development of DR (56). One study showed that overexpression and hyperphosphorylation of tau induced ferroptosis in nerve cells (57). Other studies have shown that decreased levels of GPx-4 lead to lower numbers of hippocampal neurons and astrocytes in adult mouse models of Alzheimer’s disease (58) and that cell death due to decreased GPx-4 is a characteristic of ferroptosis.

Moreover, other studies have found that oxidative stress is related to the onset and progression of DR (59). Recent studies suggest that oxidative stress and photoreceptor dysfunction in diabetic individuals may precede the early vascular pathology (60). The retina is a tissue with high oxygen consumption and, when it is in a high glucose state, antioxidants such as reduced GSH are consumed in large quantities, making it more vulnerable to damage than other organs (59). NFE2L2 is an important antioxidant. Many studies have found that activation of the NFE2L2 pathway can protect the retinal tissue of diabetic patients (61–63). Several studies of HCC cells have shown that inhibition of NFE2L2 expression can increase the anticancer activity of the chemicals erastin and sorafenib, with inhibition of NFE2L2 inducing ferroptosis and killing HCC cells (64). High glucose conditions trigger physiological and pathological conditions in tissues, together with enhanced accumulation of advanced glycation end products (AGEs), which successively cause epithelial tissue necrobiosis and lesions in retinal capillaries (65). These findings highlight key roles for human retinal endothelial cells (HRECs) in the pathophysiology of DR and suggest that these cells are as promising targets in DR therapy.

TRIM46, a gene located on chromosome 1q21 locus (66), is a very crucial member of the E3 ubiquitin tripartite motif (TRIM) family. TRIM46 may be a potential biomarker of carcinogenesis and regulates the proliferation of cancer cells *via* mediated VEGF (67). Through *in vitro* experiments, a recent study found that high glucose treatment time-dependently induced ferroptosis in HRCECs and induced TRIM46 expression (68). Overexpression of TRIM46 mediated by lentiviral strongly weakened cell resistance against ferroptosis in HRCECs in response to high glucose, whereas RNAi-mediated knockdown of TRIM46 had the opposite effect. Ferroptosis agonist RSL3 could reverse the protective effects of TRIM46 silencing. Notably, TRIM46 has interactions with GPx-4, which is an important enzyme that inhibits ferroptosis and promotes ubiquitination of GPx-4. Furthermore, overexpression of GPx-4 mediated by lentiviral reduced the impacts of TRIM46 overexpression and protected the cells against ferroptosis induced by high glucose. However, further studies will be necessary to determine the precise molecular mechanism. Hence, ferroptosis may become a new direction in the study of DR.

### Retinal Ischemia-Reperfusion Injury

RIRI, playing critical roles in the etiology of blindness, is caused by a variety of retinal vascular diseases. When the blood supply is restored to the ischemic retina, reperfusion intensifies the cell death and causes considerable damage to retinal function, which can damage retinal neurons and trigger optic ganglion cell apoptosis (69). The current treatment of damaged retinal ganglion cells and the atrophied optic nerve is not ideal. Therefore, the identification of new therapeutic targets is of considerable clinical significance (70). In recent years, it has been found that ferroptosis inhibition can effectively ameliorate ischemic injury of the brain, heart, liver, and kidney (32, 71), proving that ferroptosis is involved in the ischemic injury of vital organs in the human body. However, the retinal vasculature and cerebral vasculature share the same embryonic origin, and many similarities have been found in the pathologies affecting the two tissues. Therefore, we speculate that ferroptosis may also play an important role in RIRI. Moreover, studies have shown that the inflammatory response of RIRI can increase vascular leakage and participate in the death of retinal ganglion cells. Recent work has shown that ferroptosis is accompanied by inflammatory manifestations (63). Some inflammatory mediators present in ferroptosis can also be found in the inflammatory response of RIRI, which further supports researchers’ hypothesis, but no relevant experimental studies have been conducted so far.

### Glaucoma

Glaucoma is a type of blinding disease with characteristic optic nerve damage and a visual field defect that is mainly due to the progressive loss of retinal ganglion cells (RGCs). The survival of RGCs is tightly linked to the regulation of the iron steady state modulated by mitochondrial protein involved in iron. Frataxin (FXN) is an iron chaperone protein that enhances iron deposition within the cells mitochondria and increases Fe^2+^ readily availability (72). A sudden increase in intraocular pressure leads to an upregulation of endogenous retinal FXN. Overexpression of FXN in Müller cells protects RGCs from acute ischemia/reperfusion injury (73). The improved RGC survival is related to the maintenance of mitochondrial function and an increased antioxidant response, which is associated with iron homeostasis regulated by neurotransmission. Excess glutamate binds to the cell surface glutamate receptors NMDARs (N-methyl-D-aspartic acid receptors), leading to a toxic effect of calcium influx and ultimately causing RGC death (74).

There is emerging preliminary evidence that ferroptosis has been indicated to contribute to the death of RGC. Stimulation of NMDARs (75) induces iron deposition in cells by activating the GTP (guanosine triphosphate) -binding protein Dexrasl, which contributes to activate the metal ion transporters, including the divalent metal transporter-1 (DMT1) (23). Selective iron-chelating agents significantly reduce NMDA toxicity, and Dexrasl depletion in rats similarly reduces NMDA-induced death of RGC (23), suggesting that Dexrasl plays an important role in iron metabolism along with iron-dependent type of cell death. In this case, subsequent experimental researches confirm that glutamate receptors may play a pivotal role in death of RGC. Sakamoto et al. (76) found that intravitreal injection of NMDA led to Fe^2+^ accumulation in RGCs and triggered their apoptosis, resulting in a lower number of cells 7 days after injection. At the same time, the addition of iron-chelating agents prevented the retinal damage caused by NMDA and decreased Fe^2+^ accumulation and lipid peroxidation. The ferric iron chelators including DFO and deferasirox (DFX) can shield RGCs against excitoneurotoxicity or intraocular pressure (IOP) disorders by reducing the oxidative stress in rats (76, 77). The cellular reaction state and Fe^2+^ reciprocally act to induce a regeneration loop in RGCs.

Generally, these findings demonstrate that metal-specific metallochaperone, iron transport (e.g., DMT1), and cellular metabolism together regulate the iron physiological state. Therefore, metallochaperone dysfunction, iron import channels activation, and/or oxidative stress can lead to biometal dyshomeostasis, and resulting into a loss of RGCs. In addition, the iron particle level is clearly higher in the humor of eye disease patients than in healthy persons. There is also a suggestion that iron is a risk factor for primary open-angle glaucoma (78), which supports the hypothesis. However, many queries still ought to be resolved, such as emerging evidence of distribution on levels of metal iron in RGCs within glaucomatous eyes and the excessive supply of iron ions, as well as where they go and how they impact mitochondria. Therefore, the actual link between ferroptosis and eye disease has to be tested. Several open queries remain regarding the molecular mechanisms underlying biological processes. According to using advances at single-cell level and visualization of subcellular structures, the underlying mechanisms of ferroptosis affecting the mitochondria in neurites of RGCs within the pathogenesis of eye disease will be uncovered.

### Retinoblastoma

Retinoblastoma is a type of ocular malignancy in infants and young children that can be fatal and blinding. According to the complex pathogenesis of the disease, there is currently no permanently effective treatment against it. The pathogenesis of retinoblastoma is closely linked to a p53 gene mutation and retinoblastoma gene 1 (RB1) deletion, and these two genes have been confirmed to be involved in ferroptosis. The RB1 gene also plays an important role in liver cancer. Recent studies have shown that treatment of patients with advanced liver cancer with sorafenib mediating retinoblastoma protein can significantly improve the sensitivity of liver cancer cell death to iron and sensitize patients to the anticancer effects of sorafenib (79). Some scholars believe that ferroptosis is a natural mechanism for tumor inhibition under different biological conditions, and this tumor inhibition may represent the real physiological effect of this unique cell death mode (9). Based on the above evidence, the hypothesized role for ferroptosis may be a new direction in the study of retinoblastoma.

### Retinitis Pigmentosa

RP is caused by the mass death of rods and cones. Recently, an increasing number of researches on ferroptosis have been concluded as ferroptosis is a potential form to the pathological process of RP (23). Therapeutic intervention of a mouse model of RP with an iron chelator (desferrioxamine) reduces iron involvement in oxidative stress responses and photoreceptor degeneration with decreased retinal ferritin and lipid peroxidation levels (80). Under intense fluorescent irradiation, the use of norferriamine reduces retinal light damage and better preserves photoreceptor nuclei compared with control rats (81). In addition, in the model of rapidly progressive RP, iron chelating agents—namely, 5-(4-(2-hydroxyethyl) piperazin-1-yl (methyl)-8-hydroxy-quinoline) (VK28) and 5-(N-methyl-N-propargyaminomethyl)-quinoline-8-oldihydrochloride (VAR10303), preserve visual acuity and substantially rescue cones, indicating chelation of labile iron as a new method to treat RP (82).

These experimental results indirectly support the association between ferroptosis and damage of the photoreceptor cells, and its implication in the pathogenesis of RP. More important evidence is that the increased activity of multifunctional antioxidant enzymes protects photoreceptor cells from oxidative injury (23). Through mentioned earlier, GPx-4 is an antioxidant enzyme that is an important regulatory factor in ferroptosis. In 2009, a number of studies showed that the GPx-4 overexpression strongly protected the retina from oxidative damage-induced retinal degeneration (83). Furthermore, SOD1 overexpression in a transgenic *in vitro* model, which is an essential important component of the retinal antioxidant enzyme system, has a protective effect on the retina from severe oxidative damage (23). These studies indicate the potential role of iron regulation in the biologic therapy era for RP. Nevertheless, effects of alternative ferroptosis regulators, including FER-1 or liproxstatin-1, on the prevention or treatment of RP is still lacking. Interestingly, recent studies revealed that ferroptosis has been implicated in sodium iodate-induced ARPE-19 necrobiosis, indicating that ferroptosis participates in retinal degeneration, which provides evidence on the association of ferroptosis with RP (47, 82). The authors discovered that sodium iodate increased the pool of intracellular labile iron but decreased the levels of intracellular GSH and aminoalkanoic acid. DFO and FER-1 block ARPE-19 necrobiosis from sodium iodate exposure (47, 82). These studies support the comparatively robust association between RP and ferroptosis.

The evidence indicates that ferroptosis is closely related to the pathological process of RP. Therefore, it might be useful to analyze whether or not antiferroptotic events, such as decreased hemochromatosis, increased use of antioxidants, and reduced supermolecule peroxidation, might prevent photoreceptor from death in RP. Yet, apart from the use of iron chelators, increasing the expressions of GSH and GPx-4 while decreasing that of ACSL4 could help to explain the involvement of ferroptosis in the pathogenesis and progression of RP. However, additional studies of ferroptosis are essential. In a mouse model of RP, SOD1 deficiency leads to increased retinal oxidative damage and accelerated loss of cone function through reduced expression of SOD1 and GPx-4 (84). Therefore, ferroptosis may become a new direction for the study of RP.

### Age-Related Cataracts

Cataract is the single leading cause of blindness in developing as well as in developed countries and age is strongly linked to their development (85). Age-related cataract is generally considered to be the direct result of cumulative deposition of the crystallin proteins in lens. Risk factors for cataract vary worldwide, of which, emerging evidence suggests that cumulative deposition of ROS and disruption of redox homeostasis in lens play a very critical role in the pathogenesis and treatment of age-related cataractogenesis (75, 86, 87). The drop in the *de novo* enzymatic synthesis of GSH in the aged and cataractous human lenses especially in conjunction with the oxidation overexpression and use of GSH strongly decreases the content of lens GSH (88, 89). The formation of such barriers greatly impedes the diffusion of GSH to nucleus of the lens, which makes the nucleus more susceptible to certain types of oxidative damage and protein aggregation (90). Aging and cataractous human lens display impaired redox homeostasis, decreased GSH deposition, disrupted GPX activity, increased content on iron redox, and promoted lipid oxidation. Such characteristics match the morphological and biochemical criteria of ferroptosis. Wei et al. (91) found that lens epithelial cells were extremely liable to ferroptosis evoked by erastin, a system Xc(-) inhibitor, or RSL3, a GPX4 inhibitor from studies conducted using *in vitro* lens epithelial cell and an *ex vivo* lens epithelial tissue. This evidence shows that lens epithelial cells are particularly susceptible to ferroptosis and sensitization during biologic processes related to aging. Babizhayev et al. (92) suggested that drug administration such as intravitreal injection of lipid peroxides induces posterior subcapsular (PSC) cataract in a rabbit-eye model. The researchers highlighted the importance of lipid peroxidation in pathophysiologic cataractogenesis (93). However, no specific biomarkers allow the explicit identification of ferroptosis *in vivo*. Additional researches are needed to evaluate the mechanisms of ferroptosis for cataract formation during aging. Specifically, delineating the mechanisms of GPx-4 in cataractogenesis will be valuable to underlie the association between lipid peroxidation, ferroptosis, and cataractogenesis.

## Conclusion and Outlook

In the last decade, many researchers have devoted themselves to the study of ferroptosis, making considerable progress in all aspects of ferroptosis research. An increasing variety of researches have verified that ferroptosis plays a significant role in a very specific type of pathophysiological process. However, its complicated restrictive mechanism remains elusive. Ferroptosis may be an extremely complicated type of necrobiosis. Although ferroptosis inducers hold promising potential in the pathology of eye diseases, the function and mechanisms of ferroptosis involving these diseases has not been identified by clinical studies. This study provides an in-depth review of the latest advancements in progress in ferroptosis, with findings showing that, in addition to the various metabolic pathways such as iron ions, amino acids, and lipids, several transcription factors including NFE2L2 and p53, are linked to regulation of ferroptosis. Because previous reports have indicated that ferroptosis is crucial to eye diseases, such as RP and cataract, administration of ferroptosis inhibitors could alleviate these changes, as mentioned above. Otherwise, the molecular mechanisms underlying these inhibitors remain far from clear. Therefore, in-depth anatomic and molecular knowledge of the ferroptosis involving in the pathogenesis of eye diseases can provide novel targets and ideas for medical healthcare, prevention, and treatment of the associated diseases. Despite the outstanding progress achieved in valuable animal model for studying on ferroptosis and its association with ocular abnormalities, several problems related to the biological behavior of ferroptosis should be resolved before the basic results can be translated into clinical therapy for eye diseases.

Overall, ferroptosis is probably strongly involved in some eye diseases. However, it is not clear whether its pathological mechanisms in cell or animal models closely tally with those in people. Although the potential regulatory mechanisms and signaling pathways in ferroptosis related to eye diseases have been extensively explored, robust proof for ferroptosis in eye diseases involving human cells and human autopsy tissues continues to be limited. Currently, ferroptosis solely provides a link between organ dysfunction and the accumulation of lipid peroxidation products ascertained in human pathology. However, the mechanism by which ferroptosis regulates cell and tissue degradation continues to be unclear. Moreover, whether or not ferroptosis induces cell senescence and tissue changes in eye diseases requires additional research. Worth noting, there is no direct proof regarding the clinical effects of ferroptosis-specific inhibitors or activators in eye diseases. Therefore, in future studies (e.g., clinical trials or targeted research), ferroptosis needs to be targeted as a potential approach to provide novel therapeutic strategies for preventing, controlling, and treating eye diseases.

## Author Contributions

All authors listed have made a substantial, direct, and intellectual contribution to the work, and approved it for publication.

## Conflict of Interest

The authors declare that the research was conducted in the absence of any commercial or financial relationships that could be construed as a potential conflict of interest.

## Publisher’s Note

All claims expressed in this article are solely those of the authors and do not necessarily represent those of their affiliated organizations, or those of the publisher, the editors and the reviewers. Any product that may be evaluated in this article, or claim that may be made by its manufacturer, is not guaranteed or endorsed by the publisher.
